# Supplementary material for: Population differences in the length and early‐life dynamics of telomeres among European pied flycatchers
Source: Mol Ecol. 2021 Dec 20;31(23):5966–78. doi: 10.1111/mec.16312 (PMC9788103; doi:10.1111/mec.16312)

**Supplemental Information for:**

**Population differences in the length and early-life dynamics of telomeres among European pied flycatchers**

Tiia Kärkkäinen^a^*, Toni Laaksonen^a^, Malcolm Burgess^b,c^, Alejandro Cantarero^a,d^, Jesús Martínez-Padilla^e^, Jaime Potti^f^, Juan Moreno^d^, Robert L. Thomson^a,g,h^, Vallo Tilgar^i^, Antoine Stier^a,j^

^a^ Department of Biology, University of Turku, Turku, Finland

^b^ RSPB Centre for Conservation Science, Sandy, UK

^c^ Centre for Research in Animal Behaviour, University of Exeter, Exeter, UK

^d^ Department of Evolutionary Ecology, Museo Nacional de Ciencias Naturales (CSIC), Madrid, Spain

^e^ Department of Biological Conservation and Ecosystem Restoration, Pyrenean Institute of Ecology (CSIC), Spain.

^f^ Department of Evolutionary Ecology, Estación Biológica de Doñana (CSIC), Seville, Spain

^g^ Department of Biological Sciences, University of Cape Town, Rondebosch, South Africa

^h^ FitzPatrick Institute of African Ornithology, DST-NRF Centre of Excellence, University of Cape Town, Rondebosch, South Africa

^i^ Department of Zoology, Institute of Ecology and Earth Sciences, University of Tartu, Tartu, Estonia

^j^ Univ Lyon, Université Claude Bernard Lyon 1, CNRS, ENTPE, UMR 5023 LEHNA, F-69622, Villeurbanne, France

*Author of correspondence: tmakark@gmail.com

**Table of Contents:**

| **Table S1. Results from DNA concentration and purity quantification** | Page 2 |
| --- | --- |
| **Table S2. Population specific efficiencies and Cq-values for control gene**  **and telomere assays** | Page 3 |
| **Table S3. Results of linear mixed models explaining the effects of**  **Age class and Population on telomere length using subsets of the data** | Page 4 |
| **Figure S1. Locations of the study sites** | Page 5 |
| **Figure S2. Illustrating the telomere lengths of the same sample**  **measured both with two qPCR machines** | Page 6 |
| **Figure S3. Individual raw telomere length values** | Page 6 |
| **Figure S4. Telomere length values in different populations using subsets**  **of the data** | Page 7 |
| **Figure S5. Associations between migration distance and telomere length** | Page 8 |
| **Figure S6. Pied flycatcher chick body mass adjusted for clutch size** | Page 9 |
| **FigureS7. Change in telomere length during nestling period** | Page 10 |

**Table S1.** Results from DNA concentration and purity quantification using ND-1000-Spectrophotometer (mean ± sd). Large standard deviations for average concentration values are due to variation in tissue quantity among samples. All samples were however diluted to the concentration of 2.5 ng/µl before telomere length estimation. Three linear models (Concentration/[260/289]/[260/230] as dependent variable with Kenward-Roger approximation for degrees of freedom) were ran to test the differences among populations. Differences in DNA concentration were not statistically significant (F_5, 531_=1.02, p=0.40) while the differences in both 260/280 (F_5, 531_=2.45, p=0.03) and 260/230 (F_5, 531_=8.70, p<.0001) ratios reached statistical significance. Including the ratio-values as covariates in the statistical analyses presented in the main text with telomere length as dependent variable did not change the results or conclusion, thus these covariates were removed from the final models to reduce model parameters.

| **Population** | **DNA concentration [ng/ul] (mean ± sd)** | **260/280 (mean ± sd)** | **260/230 (mean ± sd)** |
| --- | --- | --- | --- |
| **Oulu, Finland** | 255,27 ± 208,69 | 1,93 ± 0,06 | 2,22 ± 0,25 |
| **Turku, Finland** | 231,93 ± 170,90 | 1,94 ± 0,08 | 2,26 ± 0,32 |
| **Kilingi-Nõmme, Estonia** | 209,86 ± 155,71 | 1,93 ± 0,05 | 2,23 ± 0,26 |
| **East Dartmoor, UK** | 258,94 ± 236,63 | 1,92 ± 0,04 | 2,44 ± 0,14 |
| **La Hiruela, Spain** | 260,09 ± 226,12 | 1,94 ± 0,07 | 2,30 ± 0,30 |
| **Valsaín, Spain** | 269,35 ± 218,84 | 1,92 ± 0,05 | 2,28 ± 0,24 |
| **All** | 248,64 ± 206,65 | 1,93 ± 0,06 | 2,29 ± 0,27 |

**Table S2.** Population specific (Mean ± sd) efficiencies and Cq-values for control gene (SCG) and telomere (TELO) assays. Three linear models (SCG Cq/SCG Efficiency/TELO Efficiency as dependent variable with Kenward-Roger approximation for degrees of freedom) were ran to test the differences among populations. Differences in SCG Cq-values were not statistically significant (F_5, 528_=1.48, p=0.20) while the differences in both SCG (F_5, 528_=8.11, p<.0001) and TELO (F_5, 528_=12.66, p<.0001) efficiencies reached statistical significance. Including both assay efficiencies as covariates in the statistical analyses presented in the main text with telomere length as dependent variable did not change the results or conclusion, thus these covariates were removed from the final models to reduce model parameters.

| **Population** | **SCG Efficiency [mean ± sd]** | **SCG Cq [mean ± sd]** | **TELO Efficiency [mean ± sd]** | **TELO Cq [mean ± sd]** |
| --- | --- | --- | --- | --- |
| **Oulu, Finland** | 1.87 ± 0.11 | 23.83 ± 1.50 | 1.88 ± 0.08 | 7.35 ± 1.05 |
| **Turku, Finland** | 1.91 ± 0.10 | 24.13 ± 1.44 | 1.93 ± 0.08 | 7.61 ± 0.92 |
| **Kilingi-Nõmme, Estonia** | 1.87 ± 0.11 | 23.82 ± 1.20 | 1.89 ± 0.07 | 7.40 ± 0.72 |
| **East Dartmoor, England** | 1.94 ± 0.04 | 23.98 ± 1.12 | 1.95 ± 0.06 | 7.03 ± 0.65 |
| **La Hiruela, Spain** | 1.90 ± 0.09 | 23.73 ± 1.64 | 1.89 ± 0.08 | 7.22 ± 1.12 |
| **Valsaín, Spain** | 1.88 ± 0.10 | 23.75 ± 1.06 | 1.91 ± 008 | 7.02 ± 0.75 |

**Table S3**. Results of linear mixed models explaining the effects of Age class and Population on telomere length using subsets of the whole data including samples analyzed only with a) QuantStudio or b) MicPCR

| a) |  | Telomere length | | | |
| --- | --- | --- | --- | --- | --- |
|  | Independent variable | Estimate ± se | df_num,dem_ | F ⁄ χ²* | *P* |
|  | Fixed effects |  |  |  |  |
|  | Intercept | 0.19 ± 0.14 | 114 |  |  |
|  | Age class |  | 2, 172.5 | 38.62 | < .0001 |
|  | Population |  | 5, 80.24 | 7.80 | < .0001 |
|  | Random effect |  |  |  |  |
|  | Nest box | 0.13 ± 0.04 | 1 | 13.24 | 0.0003 |
|  | ID | 0.06 ± 0.06 | 1 | 1.06 | 0.30 |
|  | qPCR plate | -0.00 ± 0.01 | 1 | 0.00 | 0.97 |
|  | Residual | 0.37 ± 0.06 |  |  |  |
| b) |  | Telomere length | | | |
|  | Independent variable | Estimate ± se | df_num,dem_ | F ⁄ χ²* | *P* |
|  | Fixed effects |  |  |  |  |
|  | Intercept | 0.51 ± 0.31 | 48.61 |  |  |
|  | Age class |  | 2, 66.75 | 16.33 | < .0001 |
|  | Population |  | 5, 39.68 | 7.26 | < .0001 |
|  | Random effect |  |  |  |  |
|  | Nest box | 0.10 ± 0.09 | 1 | 1.54 | 0.21 |
|  | ID | 0.12 ± 0.22 | 1 | 0.26 | 0.61 |
|  | qPCR plate | 0.28 ± 0.16 | 1 | 16.84 | < .0001 |
|  | Residual | 0.66 ± 0.21 |  |  |  |
|  | *F-tests were used for significance tests of fixed effects, likelihood ratio tests (χ²) with mixture distributions and one-sided p-values were used for random effects. | | | | |


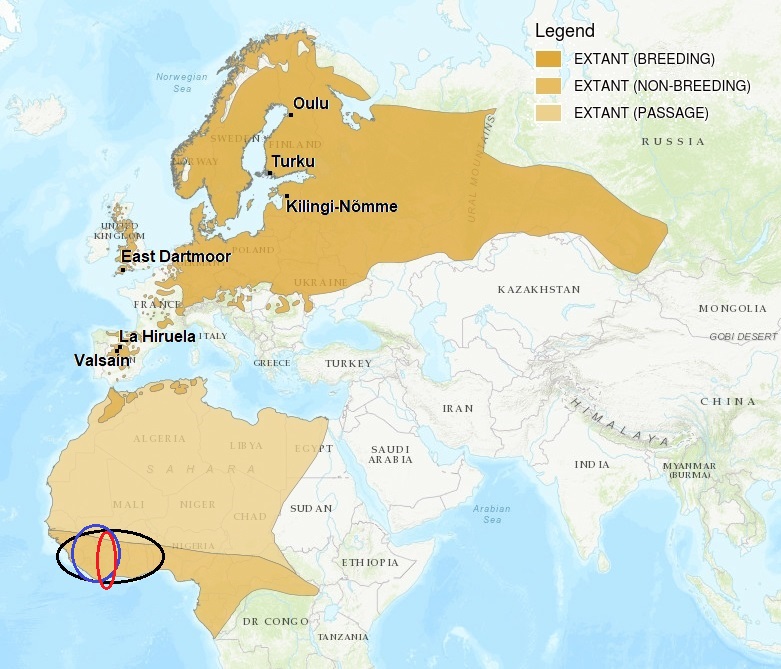
**Figure S1.** Locations of the study sites; breeding area of the pied flycatcher in Eurasia shown in orange. Birds from all populations are expected to migrate through Iberian Peninsula and west coast of Africa to their Sub-Saharan non-breeding grounds described in Ouwehand et al. 2016 (black circle; Finnish and Estonian birds blue circle; English and Spanish birds red circle). Map modified from: BirdLife International. 2018. Ficedula hypoleuca. The IUCN Red List of Threatened Species 2018: e.T22709308A131952521. https://dx.doi.org/10.2305/IUCN.UK.2018-2.RLTS.T22709308A131952521.en. Downloaded on 10 August 2021.

**Figure S2.** Illustrating the telomere lengths (T/S ratios) of the same sample measured both with QuantStudio and MicPCR. Telomere length estimates are consistently somewhat higher for MicPCR (15 out of 20 samples) accounting for somewhat low agreement repeatability of 0.851 (95% Cl [0.66, 0.94], P<0.001) between the two machines.


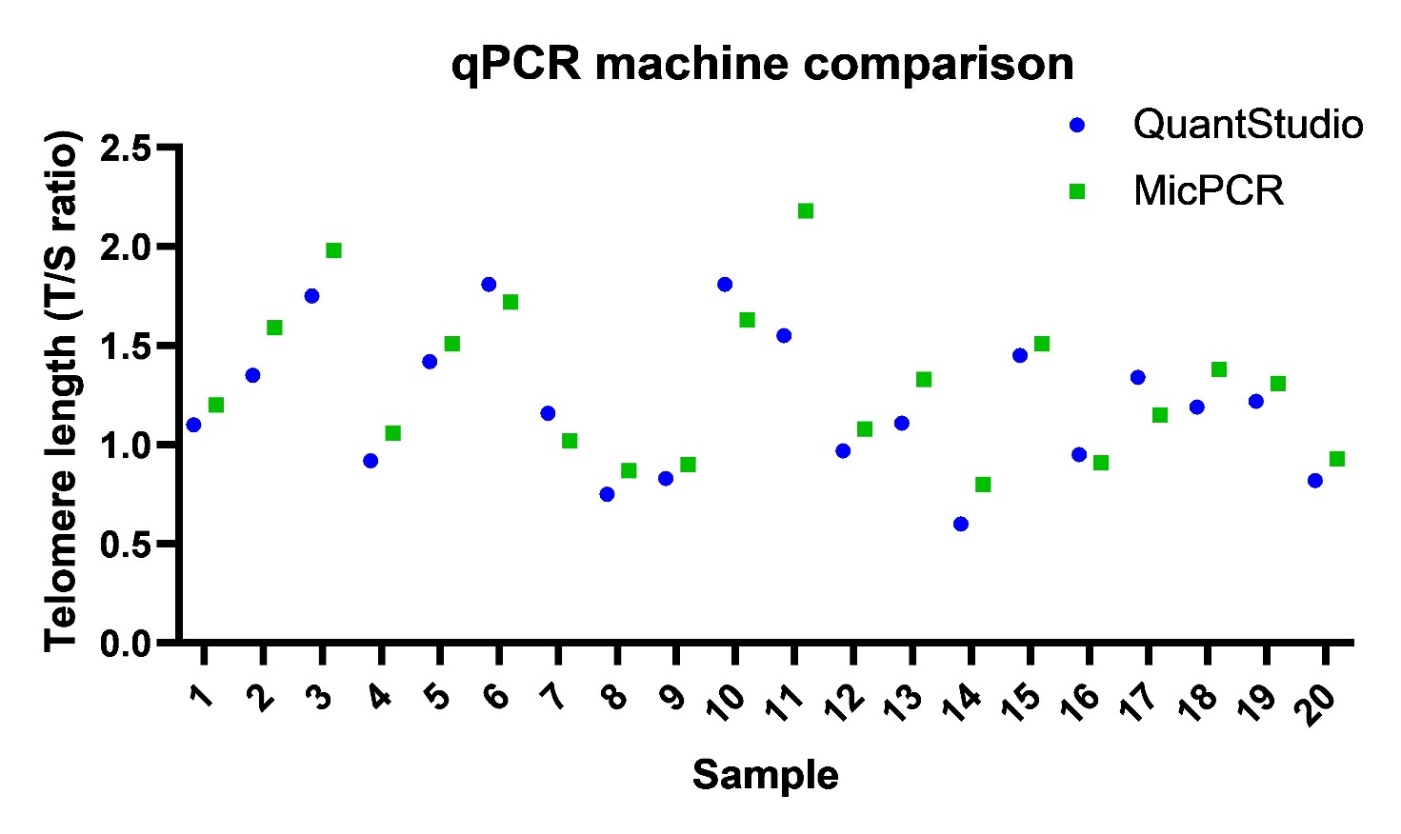


**Figure S3.** Individual raw telomere length values (T/S ratio) per population and age class. See sample sizes for Population: Nestling/Fledgling/Adult in the caption for Figure 1.


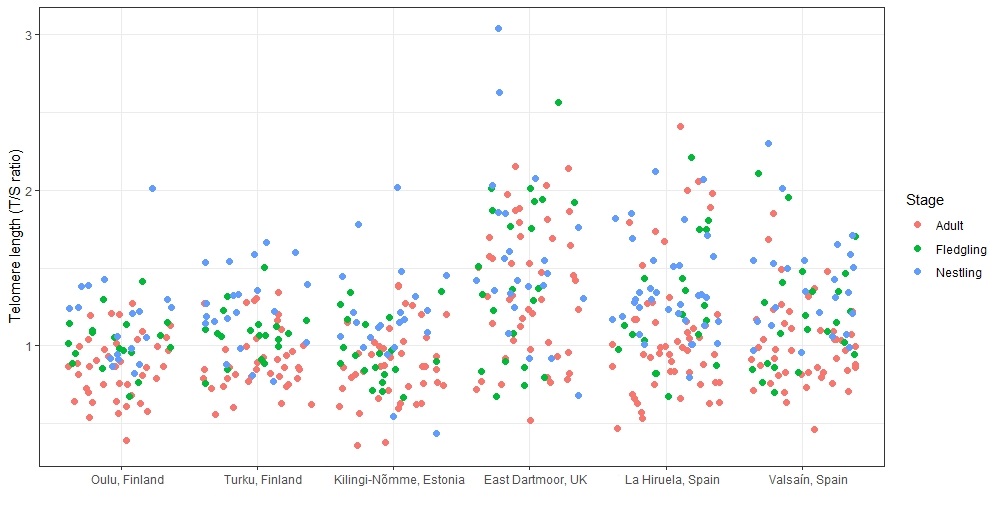


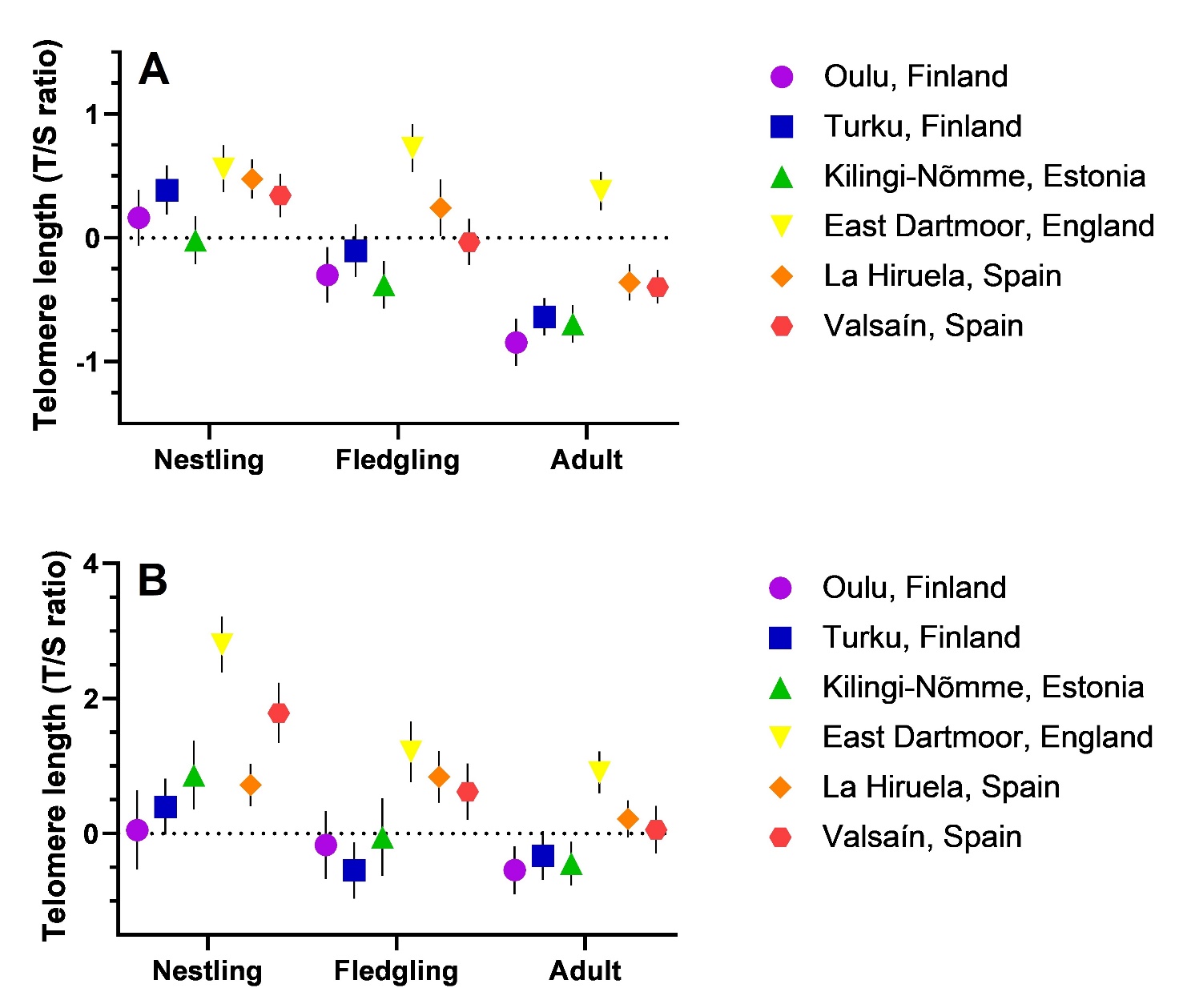
**Figure S4.** Relative telomere length in six pied flycatcher populations across a north-south gradient in Europe, from the early nestling period (Nestling; 5 days after hatching), to fledging (Fledgling; 12 days after hatching) and adulthood (Adult; end of the rearing period) using subsets of data including rTL values obtained only with a) QuantStudio, or b) MicPCR. Values are estimated marginal means based on z-scored telomere length values ± s.e.m. Sample sizes [for Population: Nestling/Fledgling/Adult] are a) Oulu: 16/15/29; Turku: 15/13/32; Kilingi-Nõmme: 18/17/31; East Dartmoor: 17/17/31; La Hiruela: 23/12/33; Valsaín: 19/17/39, and b) Oulu: 3/4/12; Turku: 6/6/9; Kilingi-Nõmme: 4/3/12; East Dartmoor: 6/5/14; La Hiruela: 12/7/19; Valsaín: 5/6/10.

**Figure S5.** Associations between migration distance (km) and relative telomere length (mean based on z-scored values) in the pied flycatcher fledglings (12 days after hatching; circles) and adults (averaged breeding pair; squares). Standard errors of the means (± sem) have been added to illustrate the population variation in telomere length. Fledgling values (circles) have been moved slightly to the right to clarify the error bars. Populations from the shortest migration distance to the longest: Spain (average of Valsaín and La Hiruela, red), England (East Dartmoor, yellow), Estonia (Kilingi-Nõmme, green), southern Finland (Turku, blue), and northern Finland (Oulu, purple).


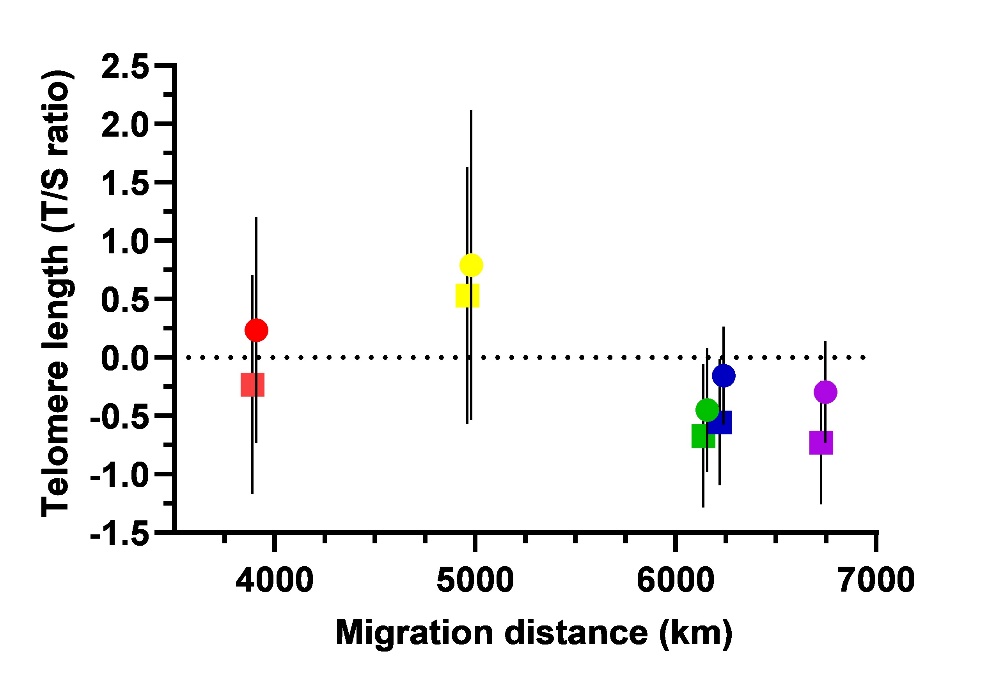


**Figure S6.** Pied flycatcher chick body mass adjusted for clutch size at day 5 (A), day 12 (B) and growth rate (Δ mass between days 12 and 5; C) in six populations across a north-south gradient in Europe. Statistically significant differences after Tukey-Kramer adjustment for multiple comparisons are indicated with different letters. Values are estimated marginal means ± s.e.m. Sample sizes [for Population: Day5/Day12/Growth] are: Oulu, Finland: 19/19/17; Turku, Finland: 21/19/18; Kilingi-Nõmme, Estonia: 22/20/19; East Dartmoor, England: 20/22/18; La Hiruela, Spain: 33/19/18; Valsaín, Spain: 24/23/21.

**
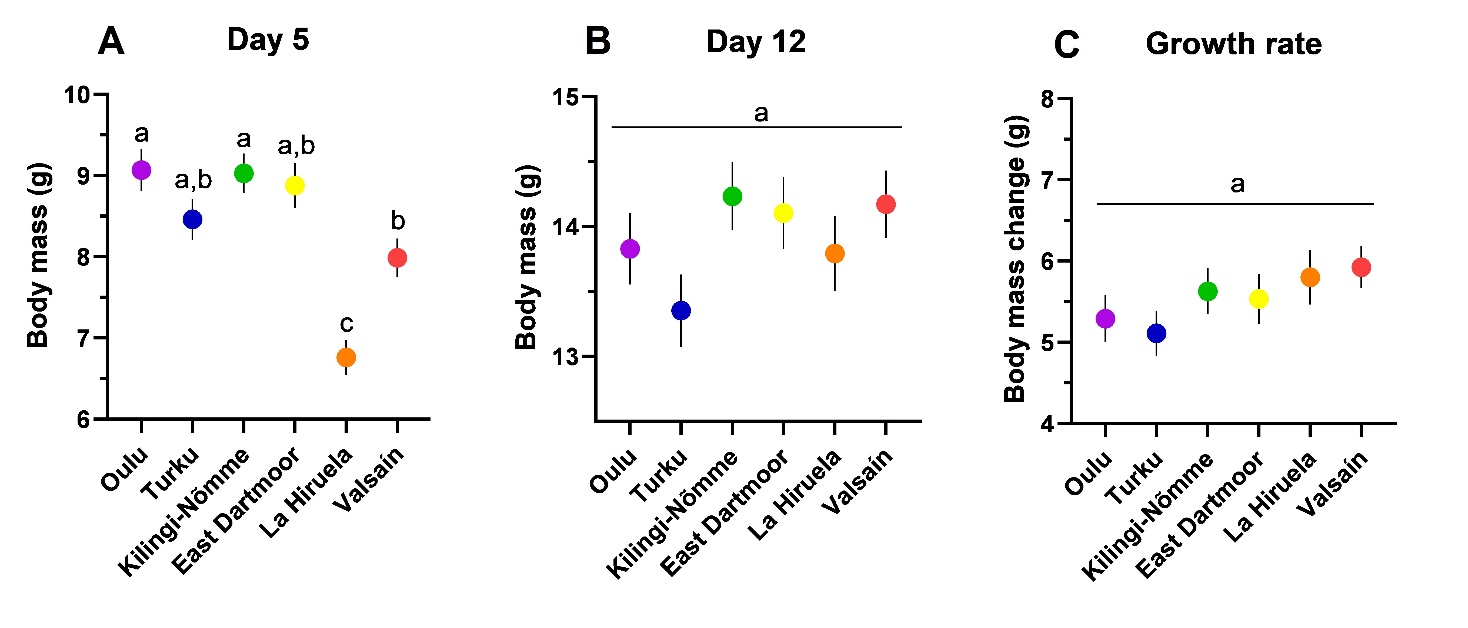
**

**Figure S7.** Change in relative telomere length during nestling period in the pied flycatcher (Δ telomere length between days 12 and 5) in six populations across a north-south gradient in Europe. The effect of population was marginally significant (*p* = 0.06) in explaining variation in early-life telomere change (see results for details). Values are estimated marginal means based on z-scored telomere length values ± s.e.m. Sample sizes [for Population] are: Oulu, Finland: 17; Turku, Finland: 18; Kilingi-Nõmme, Estonia: 19; East Dartmoor, England: 21; La Hiruela, Spain: 18; Valsaín, Spain: 21.


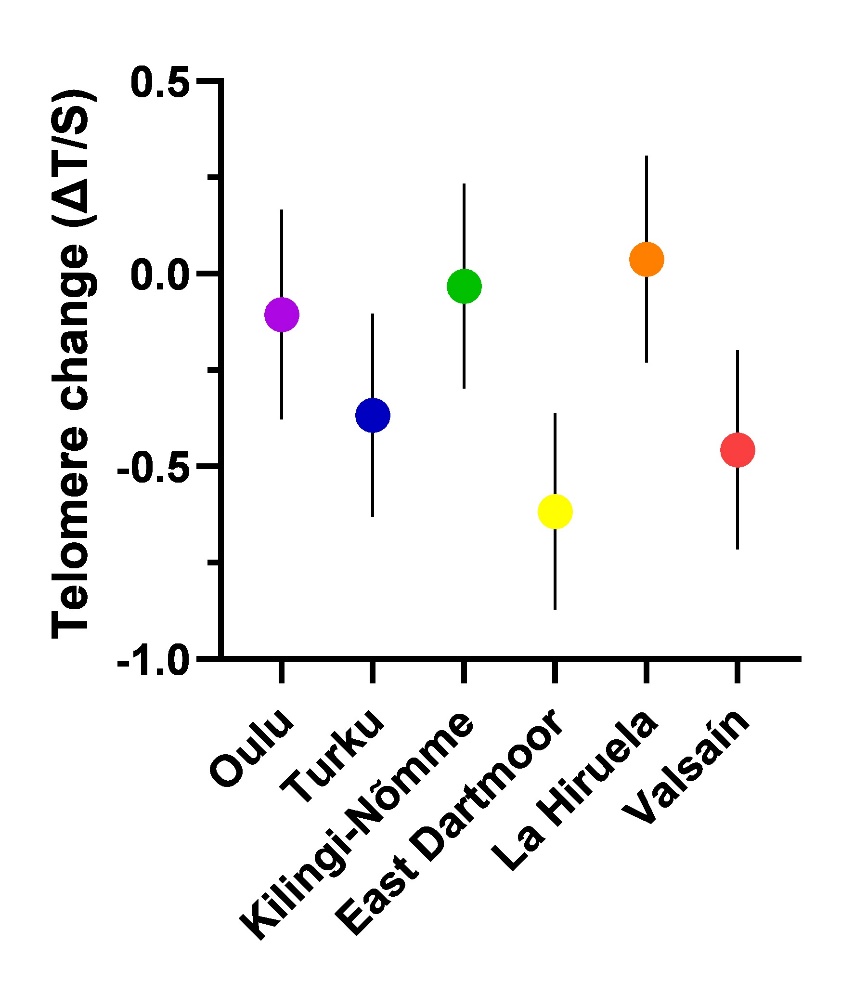

Supplement: Supplementary file 1 — App S1 [file MEC-31-5966-s001.docx]
